# Proteasome inhibition slightly improves cardiac function in mice with hypertrophic cardiomyopathy

**DOI:** 10.3389/fphys.2014.00484

**Published:** 2014-12-16

**Authors:** Saskia Schlossarek, Sonia R. Singh, Birgit Geertz, Herbert Schulz, Silke Reischmann, Norbert Hübner, Lucie Carrier

**Affiliations:** ^1^Department of Experimental Pharmacology and Toxicology, Cardiovascular Research Center, University Medical Center Hamburg-EppendorfHamburg, Germany; ^2^German Centre for Cardiovascular Research (DZHK)Hamburg/Kiel/Lübeck, Germany; ^3^Max-Delbrück-Center for Molecular Medicine (MDC)Berlin, Germany; ^4^German Centre for Cardiovascular Research (DZHK)Berlin, Germany

**Keywords:** cardiomyopathy, hypertrophic, *Mybpc3*, transgenic mice, ubiquitin-proteasome system, proteasome inhibitors

## Abstract

A growing line of evidence indicates a dysfunctional ubiquitin-proteasome system (UPS) in cardiac diseases. Anti-hypertrophic effects and improved cardiac function have been reported after treatment with proteasome inhibitors in experimental models of cardiac hypertrophy. Here we tested whether proteasome inhibition could also reverse the disease phenotype in a genetically-modified mouse model of hypertrophic cardiomyopathy (HCM), which carries a mutation in *Mybpc3*, encoding the myofilament protein cardiac myosin-binding protein C. At 7 weeks of age, homozygous mutant mice (KI) have 39% higher left ventricular mass-to-body-weight ratio and 29% lower fractional area shortening (FAS) than wild-type (WT) mice. Both groups were treated with epoxomicin (0.5 mg/kg/day) or vehicle for 1 week via osmotic minipumps. Epoxomicin inhibited the chymotrypsin-like activity by ~50% in both groups. All parameters of cardiac hypertrophy (including the fetal gene program) were not affected by epoxomicin treatment in both groups. In contrast, FAS was 12% and 35% higher in epoxomicin-treated than vehicle-treated WT and KI mice, respectively. To identify which genes or pathways could be involved in this positive effect, we performed a transcriptome analysis in KI and WT neonatal cardiac myocytes, treated or not with the proteasome inhibitor MG132 (1 μM, 24 h). This revealed 103 genes (four-fold difference; 5% FDR) which are commonly regulated in both KI and WT cardiac myocytes. Thus, even in genetically-modified mice with manifest HCM, proteasome inhibition showed beneficial effects, at least with regard to cardiac function. Targeting the UPS in cardiac diseases remains therefore a therapeutic option.

## Introduction

Along with the autophagy-lysosome pathway, the ubiquitin-proteasome system (UPS) represents the major proteolytic system of eukaryotic cells and degrades highly selectively intracellular cytosolic, nuclear and myofibrillar proteins. A main function of the UPS is to prevent accumulation of damaged, misfolded and mutant proteins, but it is also involved in a variety of biological processes such as cell proliferation, adaptation to stress and cell death (Zolk et al., 2006). The ATP-dependent proteolytic process involves polyubiquitination of the target protein through a series of enzymatic reactions and the subsequent degradation of the polyubiquitinated protein by the 26S proteasome (Mearini et al., 2008). The eukaryotic 26S proteasome itself is composed of the 19S regulatory particle, which recognizes, deubiquitinates and unfolds the target protein, and the 20S core, which degrades the target protein through three distinct proteolytic activities (chymotrypsin-like, trypsin-like, and caspase-like). These proteolytic activities can be inhibited either reversibly by peptide aldehydes and peptide boronates (e.g., bortezomib) or irreversibly by β-lactones (e.g., PS-519) and epoxyketones (e.g., epoxomicin; Kisselev et al., 2012).

Alterations of the UPS have been reported in several human and experimental cardiac diseases. Specifically, accumulation of polyubiquitinated proteins was a common feature of cardiac disorders (Kostin et al., 2003; Weekes et al., 2003; Birks et al., 2008), whereas activities of the proteasome decreased or increased depending on the status of the cardiac disease (Depre et al., 2006; Tsukamoto et al., 2006; Birks et al., 2008; Predmore et al., 2010; Schlossarek et al., 2012a). In the case of an activated UPS, partial and short-term proteasome inhibition has shown beneficial effects in different animal models. Treatment of mice with the irreversible proteasome inhibitor epoxomicin prevented both left ventricular hypertrophy (LVH) and the associated higher proteasome activity induced by short-term transverse aortic constriction (TAC; Depre et al., 2006). Similarly, low doses of the reversible proteasome inhibitor bortezomib suppressed cardiac hypertrophy in Dahl salt-sensitive rats (Meiners et al., 2008). In addition to its hypertrophy-preventing effect, proteasome inhibition has been shown to be even capable to reverse preexisting hypertrophy. Administration of epoxomicin at a stage of pronounced cardiac hypertrophy led to a stabilization of contractile parameters and a regression of preexisting hypertrophy in mice (Hedhli et al., 2008). Likewise, the irreversible proteasome inhibitor PS-519 attenuated isoprenaline-induced hypertrophy in mice (Stansfield et al., 2008). In the present study, we therefore aimed at evaluating whether epoxomicin may reduce cardiac hypertrophy and improve cardiac function in a genetically-engineered mouse model of hypertrophic cardiomyopathy (HCM), which carries a mutation in *Mybpc3*, encoding the myofilament protein cardiac myosin-binding protein C. The corresponding human c.772G>A *MYBPC3* mutation is one of the most frequent HCM mutations (found in 13% unrelated HCM patients; Olivotto et al., 2008) and associated with a bad prognosis (Richard et al., 2003). Homozygous KI mice develop systolic dysfunction after postnatal day 1 and LVH at postnatal day 3 (Gedicke-Hornung et al., 2013; Mearini et al., 2013). The present study was performed at the age of 7 weeks, an age at which KI mice have been shown to exhibit higher activities of the proteasome than WT littermates (Schlossarek et al., 2012a).

## Materials and methods

### Animals

Mice were housed in a controlled animal facility with free access to water and standard animal chow. The experimental procedures were in accordance with the German Law for the Protection of Animals and have been approved by the Authority for Health and Consumer Protection of the City State of Hamburg, Germany (Nr. 07/13). Development and initial characterization of the KI mice was previously reported (Vignier et al., 2009). Both KI and WT mice were maintained on a Black Swiss background.

### Administration of epoxomicin

Administration of epoxomicin (Enzo Life Sciences) was delivered to mice by subcutaneously implanted osmotic minipumps (Alzet, model 1007D) with a dose of 0.5 mg/kg/day for 1 week. Epoxomicin was diluted in NaCl (with 10% DMSO), control pumps delivered vehicle alone. After filling, the pumps were incubated in saline at 37°C overnight to achieve the full pumping rate from the beginning. Animals were anesthetized with isoflurane (1.5%, Abbott Inc.) for minipump implantation. No decrease in body weight, no complications and/or side effects related to treatment with epoxomicin were observed.

### Echocardiography

Transthoracic echocardiography was performed before minipump implantation and after 1-week treatment using the Vevo 2100 System (VisualSonics, Toronto, Canada). Animals were anesthetized with isoflurane (1–2%, Abbott Inc.) and fixed to a warming platform in a supine position. Anesthetic depth was monitored by electrocardiogram and respiration rate. B-mode images were obtained using a MS 400 transducer with a frame rate of 230–400 frames/s. Two-dimensional short axis views were recorded at the mid-papillary muscle level. The dimensions of the left ventricle were measured in a short axis view in diastole and systole. All images were recorded digitally and off-line analysis was performed using the Vevo 2100 software.

### Determination of the chymotrypsin-like activity

The chymotrypsin-like activity of the proteasome was assessed in ventricular cytosolic lysates as described previously (Schlossarek et al., 2012a). For determination of the activity, 30 μg of protein were diluted in incubation buffer (20 mM HEPES, 0.5 mM EDTA, 5 mM DTT, 0.1 mg/ml ovalbumin) to a final volume of 50 μl. Samples were pre-incubated in this buffer for 2 h at 4°C. Following pre-incubation, the synthetic fluorogenic substrate Suc-LLVY-AMC (Merck Biosciences) was added to the samples at a final concentration of 60 μM. After incubation in the dark for 1 h at 37°C, the fluorescence of the released AMC reporter was measured using the TECAN Safire^2^ microplate reader at an excitation wavelength of 380 nm and an emission wavelength of 460 nm. Each sample was measured in triplicate. The mean of the blank (incubation buffer only) was subtracted from the mean of each sample triplicate.

### Western blot

Western blot was performed in ventricular cytosolic lysates as described previously (Schlossarek et al., 2012b). Primary antibody was directed against the β5-subunit of the proteasome (kindly given by X.J. Wang, University of South Dakota, 1:5000). Secondary antibody was anti-rabbit (Sigma, 1:6000, peroxidase-conjugated). Signals were revealed with SuperSignal® West Dura extended duration substrate (Pierce) and acquired with the Chemie Genius^2^ Bio Imaging System.

### Analysis of ventricular mRNAs

Total RNA was extracted from mouse ventricles using the SV Total RNA Isolation Kit (Promega) according to the manufacturer's instructions. RT-qPCR was performed as described previously (Schlossarek et al., 2012b). The primers used to amplify the β5-subunit of the proteasome (*Psmb5)*, atrial natriuretic peptide (*Nppa)*, α-skeletal actin (*Acta1)*, and guanine nucleotide binding protein, alpha stimulating *(Gnas)* are listed in Table 1. *Gnas* was used as an endogenous control to normalize the quantification of the target mRNAs for difference in the amount of total RNA added to each reaction.

**Table 1:** **PCR primer names and sequences**.

**Gene name**	**Target name**	**Sequence**
*Psmb5* F	Proteasome subunit, β5	5′-GAGCTTCGCAATAAGGAACG-3′
*Psmb5* R	Proteasome subunit, β5	5′-CTGTTCCCCTCGCTGTCTAC-3′
*Nppa* F	Atrial natriuretic peptide	5′-ATCTGCCCTCTTGAAAAGCA-3′
*Nppa* R	Atrial natriuretic peptide	5′-ACACACCACAAGGGCTTAGG-3′
*Acta1* F	α-skeletal actin	5′-CCCCTGAGGAGCACCCGACT-3′
*Acta1* R	α-skeletal actin	5′-CGTTGTGGGTGACACCGTCCC-3′
*Gnas* F	Guanine nucleotide binding protein, alpha stimulating	5′-CAAGGCTCTGTGGGAGGAT-3′
*Gnas* R	Guanine nucleotide binding protein, alpha stimulating	5′-CGAAGCAGGTCCTGGTCACT-3′

*Abbreviations used are: F, forward primer; R, reverse primer*.

### Transcriptome analysis

Neonatal mouse cardiac myocytes were isolated from 0 to 4 day-old KI and WT pups and cultured as described previously (Vignier et al., 2009). After 3 days of plating cardiac myocytes were either kept untreated or treated with 1 μM MG132 (Calbiochem) for 24 h. Total RNA was then extracted from cultured cardiac myocytes using RNAzol® (WAK-Chemie), according to the manufacturers' instructions. RNA concentration, purity and quality were analyzed using the NanoDrop® ND-1000 spectrophotometer (Thermo Scientific). Transcriptome analysis of all samples was performed in triplicate using Illumina Beadchips (Mouse WG-6 v2.0). The wet lab methods included amplification and fragmentation of the samples, sample hybridization to the beadchip, and washing, staining and scanning of the beadchip. For analysis, the Illumina Mouse WG-6 v2.0 array data were quantile normalized on probe level (45,281 probes) without background correction using Illumina GenomeStudio V2011.1. Data of the 12 samples (four conditions, three biological replicates) have been log_2_-transformed after an offset addition (16). Probes fail to underrun a detection *p*-value of 0.05 in any sample have been discarded before test statistic (25,080 probes left). Probes and samples were analyzed on significant expression differences according to the genotype (KI or WT) and treatment (MG132-treated or untreated) using Two-Way ANOVA statistic followed by FDR multiple testing corrections (Benjamini and Hochberg, 1995). To avoid batch effects, the Illumina slide was used as a cofactor in the test statistic. Probes which undergo 5% FDR were selected as differentially expressed. Probes, significant and more than four-fold regulated between any of the four conditions, were clustered and heatmap visualized. We performed hierarchical Euclidean average linkage clustering for initial expression profile visualization (**Figure 5**) using the pheatmap R package. Protein interaction of genes regulated by MG132-treatment have been further investigated by using the String platform (Snel et al., 2000) and a high confidence score (0.7).

### Statistical analysis

Data were expressed as mean ± s.e.m. Statistical analyses were performed by Two-Way ANOVA followed by Bonferroni's post-test, or by paired or unpaired Student's *t*-test as indicated in the figure legends. All analyses were realized using the commercial software GraphPad Prism5 (Software Inc.). A value of *P* < 0.05 was considered statistically significant. For transcriptomic analysis, statistics are explained in the corresponding paragraph above.

## Results

### Reduction of chymotrypsin-like activity by 50% with epoxomicin

**Figure 1:**
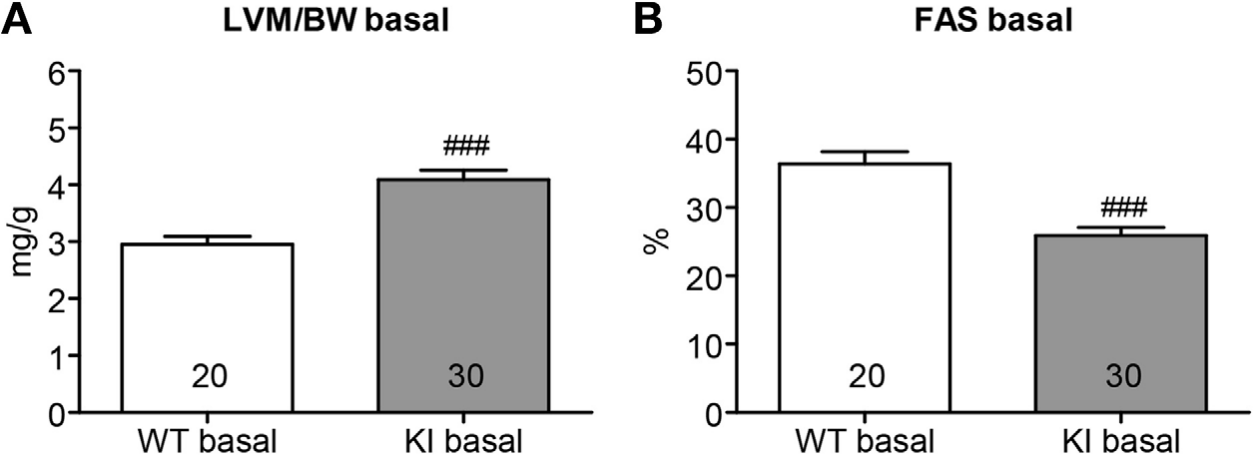
**Echocardiographic analysis in KI vs. WT mice under basal conditions**. Echocardiographic analyses were performed in KI (gray) and WT (white) mice under basal conditions, i.e., before minipump implantation. **(A)** Left ventricular mass-to-body-weight ratio (LVM/BW). **(B**) Fractional area shortening (FAS). Data are expressed as mean ± s.e.m. with ^###^*P* < 0.001 vs. WT, unpaired Student's *t*-test. The number of animals is indicated in the bars.

**Figure 2:**
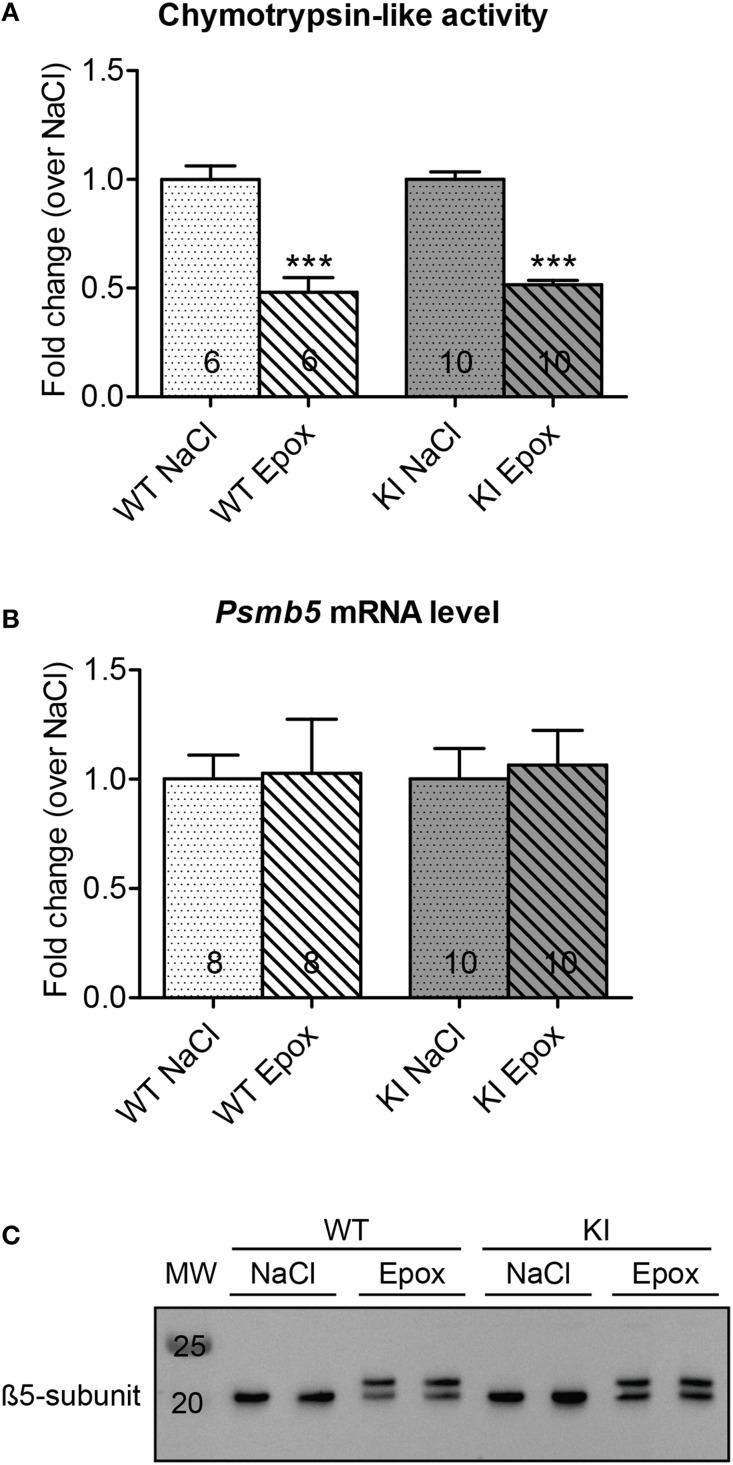
**Evaluation of chymotrypsin-like activity and β5-subunit after proteasome inhibition in KI vs. WT mice**. Analyses were performed in KI (gray) and WT (white) mice treated for 1 week with epoxomicin (Epox; stripes) or sodium chloride (NaCl; dots) via osmotic minipumps. **(A)** Chymotrypsin-like activity in ventricular cytosolic protein extracts. **(B**) β5-subunit mRNA level determined by RT-qPCR with SYBR Green and normalized to *Gnas*. **(C**) Representative Western blot stained with an anti-β5-subunit antibody. Data are expressed as mean ± s.e.m. with ^***^P < 0.001 vs. corresponding NaCl-treated group, unpaired Student's *t*-test. The number of animals is indicated in the bars.

Seven week-old KI mice presented a 39% higher left ventricular mass-to-body-weight (LVM/BW) ratio and a 29% lower fractional area shortening (FAS) than age-matched WT mice (Figure 1). We previously reported higher proteasome activities in 7-week-old KI than WT mice (Schlossarek et al., 2012a). Sex-matched KI and WT mice were treated for 1 week with epoxomicin (0.5 mg/kg/day) or vehicle (NaCl in 10% DMSO) according to Hedhli et al. (2008). Epoxomicin is a specific irreversible inhibitor of the β5-subunit of the proteasome and responsible for the chymotrypsin-like activity (Hedhli and Depre, 2010). The chymotrypsin-like activity in cytosolic protein lysates was ~50% lower in KI and WT mice treated with epoxomicin than in NaCl-treated mice (Figure 2A). Inhibition of the β5-subunit did not come along with changes in the *Psmb5* transcript level (Figure 2B), but with the appearance of two β5-subunit bands in Western blot (Figure 2C). This was already previously observed (Vignier et al., 2009) and likely represents the free (lower band) and the epoxomicin-bound (upper band) β5-subunit.

### No regression of left ventricular hypertrophy by proteasome inhibition

**Figure 3:**
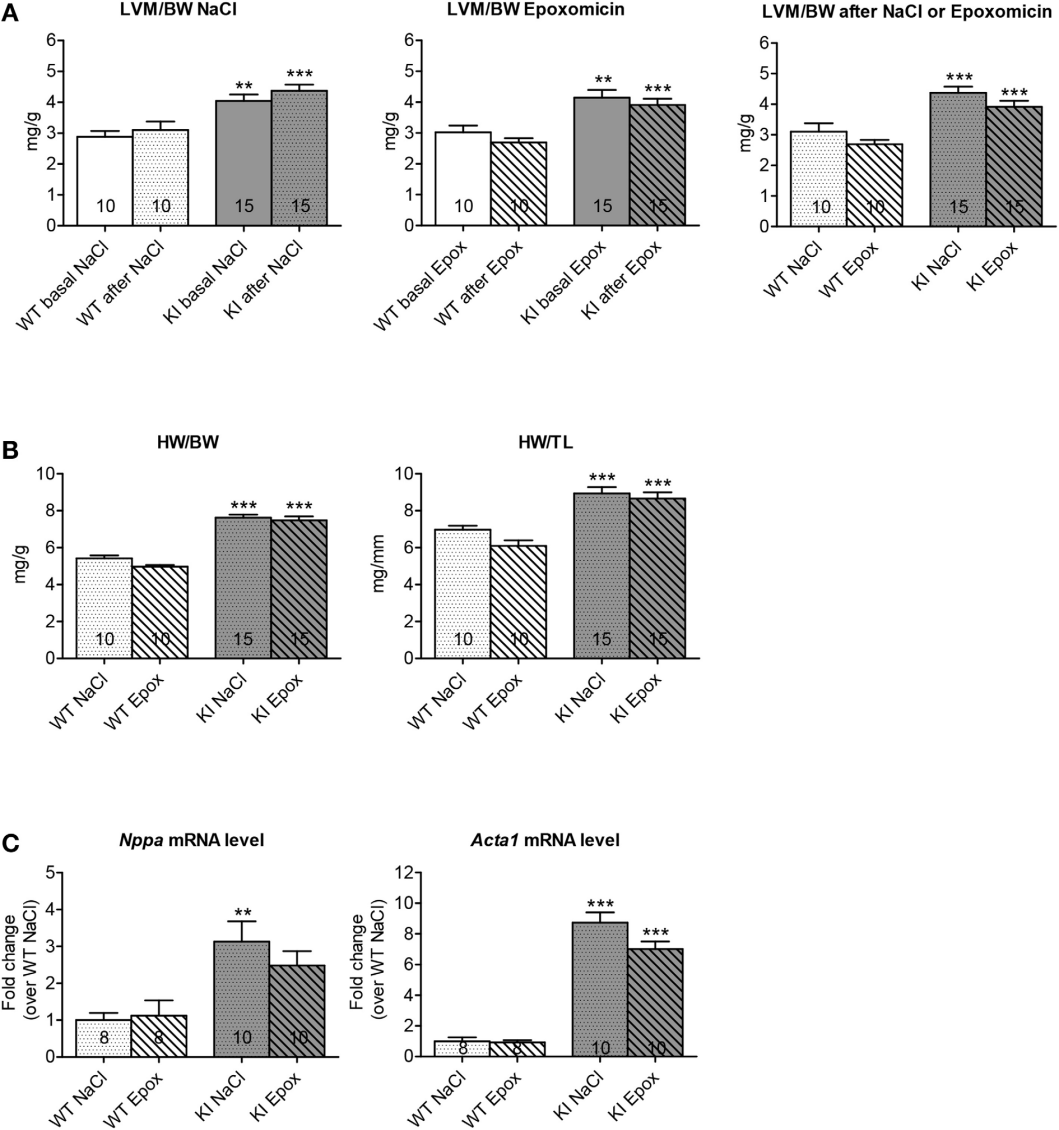
**Evaluation of cardiac hypertrophy after proteasome inhibition in KI vs. WT mice**. Analyses were performed in KI (gray) and WT (white) mice under basal conditions (basal; plain) or after 1 week of treatment with epoxomicin (Epox; stripes) or sodium chloride (NaCl; dots) via osmotic minipumps. **(A**) Left ventricular mass-to-body-weight ratio (LVM/BW) determined by echocardiography. **(B**) Heart-weight-to-body-weight ratio (HW/BW) and heart-weight-to-tibia-length ratio (HW/TL). **(C**) mRNA levels of hypertrophy markers determined by RT-qPCR with SYBR Green and normalized to *Gnas*. Data are expressed as mean ± s.e.m. with ^**^P < 0.01 and ^***^P < 0.001 vs. WT in the same condition, Two-Way ANOVA plus Bonferroni's post-test. The number of animals is indicated in the bars. Abbreviations: *Acta1*, α-skeletal actin; *Nppa*, atrial natriuretic peptide.

To determine whether proteasome inhibition had an effect on LVH, echocardiography was performed after 1 week of treatment. As expected, administration of NaCl did not alter the LVM/BW ratio in both WT and KI mice (Figure 3A, left panel). Likewise, the LVM/BW ratio did not differ before and after epoxomicin treatment in both groups (Figure 3A, middle panel). Finally, no difference in LVM/BW ratio was observed when comparing the data only after treatment in both WT and KI mice (Figure 3A, right panel). The heart-weight-to-body-weight (HW/BW) and heart-weight-to-tibia-length (HW/TL) ratios were 41% and 28% higher in NaCl-treated KI than WT mice, respectively. Epoxomicin treatment did not affect both ratios in both genotypes (Figure 3B). Finally, the transcript level of two classical hypertrophy markers *Nppa* and *Acta1* were higher in NaCl-treated KI than WT mice and were not affected by epoxomicin treatment (Figure 3C).

### Slight improvement of cardiac function by proteasome inhibition

**Figure 4:**
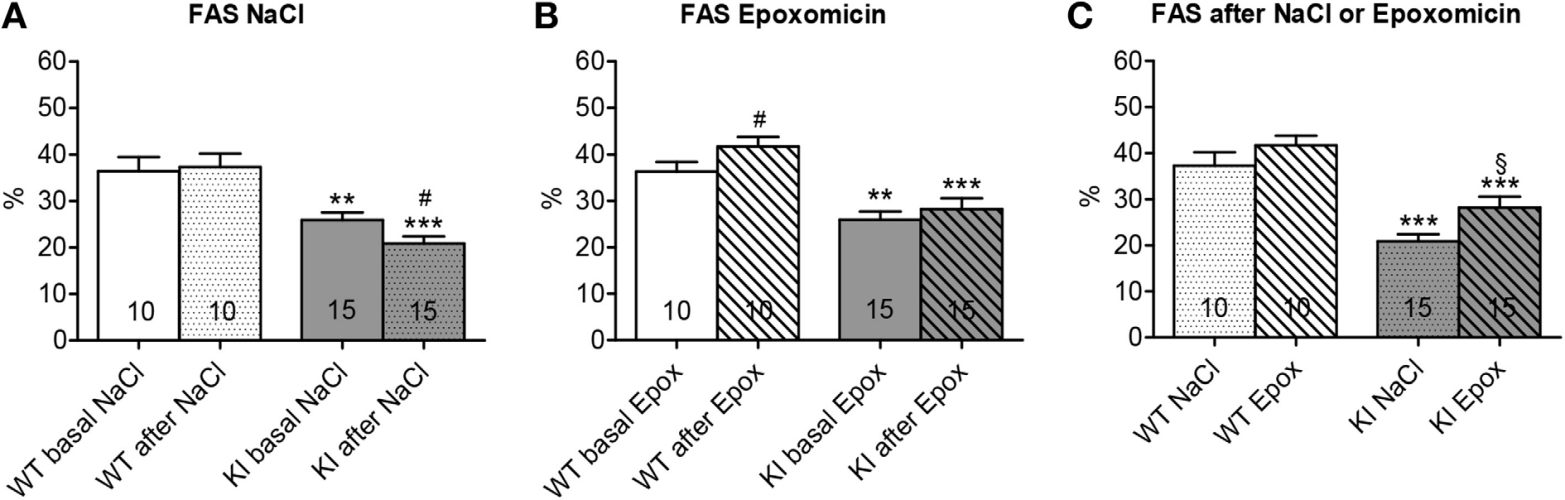
**Evaluation of cardiac function after proteasome inhibition in KI vs. WT mice**. Fractional area shortening (FAS) was determined by echocardiography in KI (gray) and WT (white) mice under basal conditions (basal; plain; **A, B**) or after 1 week of treatment with epoxomicin (Epox; stripes; **B, C**) or sodium chloride (NaCl; dots; **A, C**) via osmotic minipumps. Data are expressed as mean ± s.e.m. with ^**^P < 0.01 and ^***^P < 0.001 vs. WT in the same condition, Two-Way ANOVA plus Bonferroni's post-test, ^#^*P* < 0.05 vs. corresponding basal, paired Student's *t*-test, and ^§^*P* < 0.05 vs. KI NaCl, unpaired Student's *t*-test. The number of animals is indicated in the bars.

Cardiac function was examined by echocardiography after 1 week of treatment. FAS was not affected in WT mice, whereas it was reduced by 19% in KI mice by NaCl treatment (Figure 4A). Conversely, FAS was increased by 15% in WT, but not affected in KI mice by epoxomicin treatment (Figure 4B). However, when we compared the data after treatment, FAS was 35% higher after epoxomicin than after NaCl administration in KI mice (Figure 4C). Although the FAS did not reach the level found in WT mice, these data suggest that epoxomicin partially rescued the cardiac function in KI mice.

### Marked and significant differential gene expression in cardiac myocytes after proteasome inhibition

**Figure 5:**
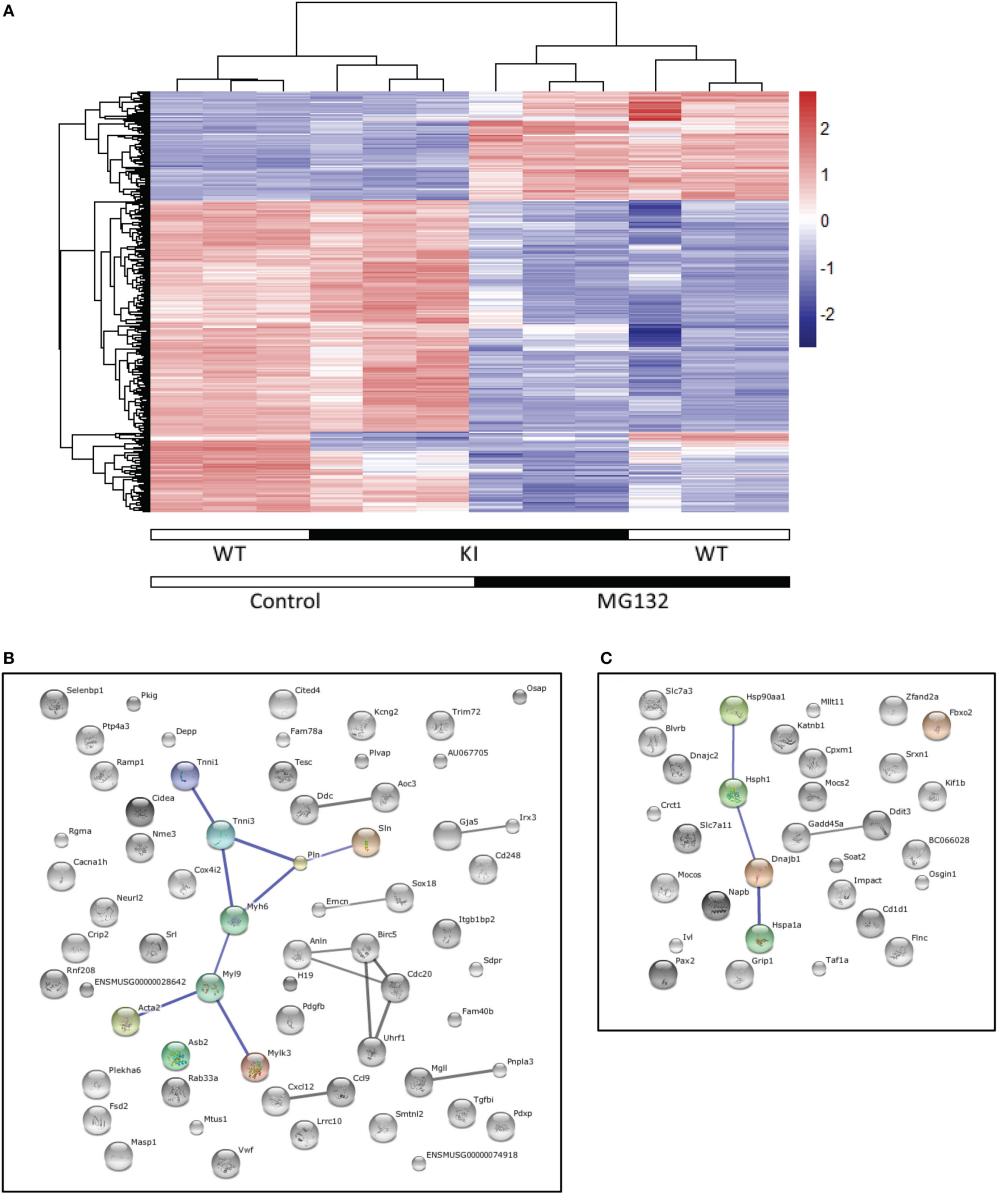
**Transcriptome analysis**. Cardiac myocytes were isolated from neonatal KI and WT mice and treated with the proteasome inhibitor MG132 (1 μM) or left untreated for 24 h. RNAs were subjected to transcriptome analysis using the Illumina Beadchips. **(A)** Clustering of the four-fold and significantly (5% FDR) regulated expressed probes (*N* = 404). The heatmap and the dendrograms representing the results of the hierarchical clustering of standardized log2 signal values. **(B), (C)** String networks of four-fold and significant differentially expressed (5% FDR) genes commonly regulated in KI and WT cardiac myocytes [*N* = 103, 68 down **(B)** and 35 up **(C)**]. Known and predicted protein interactions including direct (physical) and indirect (functional) associations are visualized in a network. The size of the connecting lines represents the impact of the connection. Only high confidence String interactions are shown.

To assess whether specific genes or biological pathways could be involved in the improved cardiac function, we performed a transcriptome analysis in WT and KI neonatal cardiac myocytes, treated or not with 1 μM of the reversible proteasome inhibitor MG132 for 24 h. Gene expression profiles were then evaluated by the Illumina Beadchips. ANOVA analysis over 25,080 expressed of 45,281 total probes led to a set of 4639 differential expressed probes (5% FDR) in strain (KI vs. WT; *N* = 37) or treatment (untreated vs. MG132-treated; *N* = 4633). No significant strain × treatment interaction was found. According to the pronounced treatment effects we further focused on markedly regulated probes (5% FDR in strain or treatment and a four-fold regulation between any pair of conditions). Hierarchical clustering of the resulting set of 404 probes (representing 339 genes) confirmed the dominance of MG132 treatment effects (Figure 5A). A total of 197 genes (141 down and 56 up) and 161 genes (113 down and 48 up) were regulated by MG132 treatment (four-fold, 5% FDR) in WT and KI cardiac myocytes, respectively. Out of them, 103 genes were commonly regulated in both groups (68 down and 35 up). Figures 5B,C show the string network of interaction of most of the genes commonly ≥four-fold down- or up-regulated in both KI and WT. Markedly down-regulated genes encode for example proteins involved in the UPS [such as ankyrin repeat and SOCS box containing 2 (ASB2) or tripartite motif-containing 72 (TRIM72)], myofilament function [such as α-myosin-heavy chain (MYH6) or cardiac troponin I (TNNI3)], or calcium handling (such as phospholamban, PLN). Markedly up-regulated genes encode for example transcription factors [such as paired box 2 (PAX2) or DNA-damage-inducible transcript 3 (DD1T3)], or molecular chaperones [such as heat shock protein 90 kDa alpha (HSP90AA1), heat shock 70 kDa protein 1a (HSPA1A) or F-box protein 2 (FBXO2) involved in protein refolding]. Details for the 404 markedly and significantly regulated probes are in the Supplemental Table 1.

## Discussion

In the present study we evaluated the cardiac phenotype after 1 week of proteasome inhibition in adolescent homozygous *Mybpc3*-targeted KI and corresponding WT mice. At the investigated age of 7 weeks, KI mice exhibited a manifest LVH and cardiac dysfunction. Based on previous publications (Hedhli et al., 2008; Stansfield et al., 2008), we hypothesized that inhibition of the UPS may be able to reduce preexisting LVH and improve cardiac function in KI mice. The major findings of the present study are: (1) no regression of preexisting hypertrophy in KI mice, (2) slight improvement of cardiac function in WT mice, and (3) a partial, but significant improvement of cardiac function in KI mice by epoxomicin treatment. Finally, MG132 treatment of cardiac myocytes revealed 103 genes which are commonly regulated in KI and WT.

Elevated or impaired UPS function has been regularly reported in experimental models of HCM and heart failure (Depre et al., 2006; Tsukamoto et al., 2006; Schlossarek et al., 2012a), suggesting that the regulation of the UPS belongs to important adaptations in cardiac disease. In this context, proteasome inhibitors have been suggested to be associated with anti-hypertrophic effects and improved cardiac function. However, the *in vivo* effect of proteasome inhibition was so far only investigated in animal models with cardiac hypertrophy induced by isoprenaline, high salt diet or TAC, but not in genetically-modified HCM mouse models as it is the case in the present study. In contrast to the previous studies, we did not observe a regression of preexisting cardiac hypertrophy after proteasome inhibition in KI mice. Neither LVM/BW nor HW/BW or HW/TL changed after 1 week of treatment with epoxomicin. Furthermore, mRNA levels of hypertrophy markers did not decrease after treatment. The discrepancy between previous and present findings may be explained by the manifestation of cardiac hypertrophy in the investigated models. In the previous studies cardiac hypertrophy was achieved in mice by 2-week of either TAC (Hedhli et al., 2008) or isoprenaline infusion (Stansfield et al., 2008). In contrast, the KI mice already develop LVH at postnatal day 3 (Gedicke-Hornung et al., 2013) and proteasome inhibition was performed at the age of 7 weeks. Therefore, cardiac hypertrophy was present for a longer time period than in previous studies and could be associated with more remodeling of the cardiac tissue.

Analysis of the cardiac function, on the other hand, displayed already challenging findings after 1 week of treatment. First, and unexpectedly, KI, but not WT mice exhibited a slight, but significant decrease in cardiac function after 1 week of NaCl administration (Figure 4A). The reason is not certain but it could be related to the combination of stress due to minipump implantation plus mutation in KI mice. This is in agreement with our previous data obtained in heterozygous KI mice (Schlossarek et al., 2012b). The stress induced by minipump implantation does not affect the WT mice, likely because they do not have a disease phenotype. Second, epoxomicin treatment improved cardiac function in WT, but not at first glance in KI mice (Figure 4B). Since the WT mice are able to cope with the stress, we directly see the effect of epoxomicin. On the contrary, since the KI mice are not able to cope with the stress (negative effect on FAS), the positive effect of epoxomicin cannot be seen directly (=no change in FAS). However, directly comparing the effects of epoxomicin and NaCl after 1 week of treatment suggests that epoxomicin indeed had a positive effect also in KI mice (Figure 4C).

To identify which genes or pathways could be involved in this positive effect, transcriptome analysis was performed in KI and WT neonatal cardiac myocytes, treated or not with the proteasome inhibitor MG132. This revealed marked and significant differentially expressed genes. Focus on the commonly regulated genes in MG132-treated KI and WT cardiac myocytes revealed up-regulation of genes encoding transcription factors or proteins involved in protein re-folding, as well as down-regulation of genes encoding proteins involved in the UPS or calcium handling. The latter for example includes PLN, which is an inhibitor of the sarcoplasmic reticulum calcium-ATPase, and therefore its down-regulation could contribute to the better cardiac function observed upon epoxomicin treatment *in vivo*.

Since the UPS plays a role in many fundamental biological processes, targeting this system for therapy is complex and particularly some conflicting data exist that argue against the anti-hypertrophic and function-stabilizing effects of proteasome inhibitors in chronic application (Herrmann et al., 2013). In addition, while bortezomib (reversible proteasome inhibitor) is generally well tolerated by patients with multiple myeloma, it has been shown to increase the occurrence of cardiac complications ranging from arrhythmia to congestive heart failure in elderly patients or patients with preexisting cardiac problems (Enrico et al., 2007; Hacihanefioglu et al., 2008; Bockorny et al., 2012).

In conclusion, whereas chronic proteasome inhibition may give additional stress to the heart and promote transition to heart failure, our data revealed that partial and short-term proteasome inhibition does not suppress LVH but slightly improved cardiac function in a mouse model of HCM. Targeting the UPS acutely therefore remains a therapeutic option for cardiac diseases with reduced cardiac function.

## Author contributions

Saskia Schlossarek, conception and design of research, management of the mouse cohorts, execution of experiments, analysis and interpretation of data, figure preparation, drafting of the manuscript. Sonia R. Singh, isolation and treatment of cardiac myocytes, execution of experiments. Birgit Geertz, recording and analysis of the echocardiographic data. Herbert Schulz, transcriptome analysis. Silke Reischmann, execution of experiments. Norbert Hübner, transcriptome analysis. Lucie Carrier, conception and design of research, analysis and interpretation of data, drafting of the manuscript. All authors critically discussed the results, and reviewed and approved the manuscript before submission.

### Conflict of interest statement

The authors declare that the research was conducted in the absence of any commercial or financial relationships that could be construed as a potential conflict of interest.

## Supplementary material

Supplemental Table 1 **Results of the descriptive and test statistic of 404 significant expressed probes are documented**.
